# Borderline Personality Disorder Diagnoses in Facial Plastic Surgery: A Large Database Analysis

**DOI:** 10.1002/oto2.70135

**Published:** 2025-05-29

**Authors:** F. Jeffrey Lorenz, Cheng Ma, Alyssa K. Givens, Scott G. Walen

**Affiliations:** ^1^ Department of Otolaryngology–Head and Neck Surgery Penn State Hershey Medical Center Hershey Pennsylvania USA

**Keywords:** borderline personality disorder, facial plastic surgery, type B personality disorder

## Abstract

**Objective:**

To determine the prevalence of borderline personality disorder among patients who undergo facial plastic surgery and identify associated demographics, clinical characteristics, and outcomes.

**Study Design:**

Retrospective cohort.

**Setting:**

More than 80 health care organizations across the United States.

**Methods:**

This retrospective cohort study queried the TriNetX Research Network to identify patients who underwent facial plastic surgeries during 2012 to 2023. Demographics, clinical characteristics, and outcomes were compared between patients with and without a diagnosis of borderline personality disorder.

**Results:**

Among 60,792 patients, there were 309 (0.51%) with a diagnosis of borderline personality disorder (mean age 45.0; 77% female, 22% male) and 60,453 controls (mean age 54.7; 63.4% female, 34.5% male). Patients with borderline personality disorder were younger (*P* < .001) and more likely to be female (*P* < .001). They were more likely to undergo rhinoplasty (*P* < .001), but less likely to have blepharoplasty (*P* < .001) or facelift (*P* = .01). They also had higher rates of psychiatric and substance use disorders (*P* < .001). Patients with borderline personality disorder were at greater risk of postoperative emergency department visits (15.9% vs 4.8%) and hospitalization (12.0% vs 6.6%) compared to controls in the first 3 months postoperatively (*P* < .001). However, these rates did not represent a significant increase relative to their own baseline levels (15.9% for emergency visits and 3.9% for inpatient admissions over a comparable 3‐month period, *P* = 1.0 and .44, respectively).

**Conclusion:**

Patients with borderline personality disorder are more likely to be younger, female, undergo rhinoplasty, have additional psychiatric comorbidities, and present to the hospital at baseline and during the recovery period.

Facial plastic surgery includes a range of procedures designed to enhance esthetic appearance, with the end goal of achieving patient satisfaction. This relies on the surgeon's attention to the psychological needs of the patient before and after the operation, a factor that many believe is as important as the surgical procedure itself.^1^ Overall, outcomes after facial plastic surgery are overwhelmingly positive, and patient satisfaction is high.^2^, ^3^, ^4^ However, there exists a small subgroup of patients who remain dissatisfied despite achieving objectively good results from the surgeon's perspective. During consultation, assessing patients who may be predisposed to disappointment with their result is important for optimizing outcomes for the patient and surgeon alike. Several factors linked to patient dissatisfaction include younger age, male gender, unrealistic expectations, and prior unsatisfactory cosmetic surgeries. Additionally, a history of depression, anxiety, personality disorders, or body dysmorphic disorder (BDD) have also been implicated.^4^, ^5^, ^6^

Borderline personality disorder (BPD) is a mental health condition classified under type B personality disorders. Individuals with BPD often act impulsively, have tumultuous interpersonal relationships driven by splitting and fear of abandonment, and have a propensity for self‐injurious behaviors.^7^, ^8^ It is characterized by significant instability in moods, behaviors, functioning, and self‐image,^9^ which is what may frequently lead these patients to facial plastic surgery clinics. It has been estimated to have a prevalence of 1.7% in the general population^8^ and may be as high as 9% in patients seeking cosmetic facial plastic surgery.^10^ Morioka and Ohkubo described that patients with BPD seek esthetic plastic surgery for two main reasons: (1) insatiable requests for esthetic procedures or (2) treatment for self‐injury.^11^

BPD presents unique challenges in the context of facial plastic surgery due to the potential for postoperative dissatisfaction and complications. Understanding the psychological profile of surgical candidates is crucial, as operating on poor surgical candidates may lead to worsening psychiatric symptoms,^12^ increased likelihood of seeking additional procedures, refund demands, or even litigation.^13^, ^14^, ^15^ Identifying patients with BPD during consultation and exploring their motivations and expectations enables surgeons to deliver patient‐centered care, ultimately fostering better outcomes for both the patient and surgeon.

In this large database study, we conducted an in‐depth analysis of BPD in the context of facial plastic surgery. We compared patients with BPD to those without, focusing on surgical, demographic, and clinical characteristics, as well as differences in outcomes and complications, based on diagnosis (International Statistical Classification of Diseases, Tenth Revision [ICD‐10]) and procedure (Current Procedural Terminology [CPT]) codes. These findings aim to enhance surgeons' preoperative evaluations and aid in identifying patients who may benefit from additional counseling when determining their surgical candidacy.

## Methods

This was a retrospective cohort study with data obtained from the TriNetX Research Network (Cambridge, MA). TriNetX is a global health research network that aggregates deidentified patient data from a consortium of more than 80 health care organizations (HCOs) across the United States.^16^ The platform includes demographic information, diagnoses, procedures, medications, lab results, and other clinical data derived from electronic health records. TriNetX ensures patient privacy and data security through deidentification, and therefore it is compliant with the Health Insurance Portability and Accountability Act (HIPAA). The Penn State Institutional Review Board deemed STUDY00018629 exempt from review as it involved the analysis of deidentified data.

The database was queried utilizing ICD‐10 and CPT codes to identify patients who underwent common facial plastic surgeries during 2012 through 2023. Surgical interventions included rhinoplasty, rhytidectomy, blepharoplasty, brow lift, and lipectomy. To ensure high‐quality data and follow‐up, patients were required to have active records for at least 1 year before and 1 year following their surgery.

Among the cohort who underwent facial plastic surgery, individuals were categorized based on whether they had a documented diagnosis of BPD (inclusive of patients diagnosed before and after their index surgery), enabling comparative analyses between those with and without BPD. Demographic variables analyzed included age, sex, region, and marital status, whereas race and ethnicity were excluded from analyses to avoid the limitations and biases often associated with these variables in database studies.^17^, ^18^ Clinical characteristics examined included the type of surgical intervention, coexisting medical conditions, psychiatric comorbidities, and substance use disorders. Postoperative outcomes such as rehospitalization and general complications within the first 3 months were also assessed, as well as early revision surgeries within the first year postsurgery. Baseline hospitalization rates were also evaluated during a 3‐month period occurring 9 to 12 months before the index facial plastic surgery to serve as a comparative reference. This time frame was intentionally selected to avoid the perioperative period and better reflect true baseline hospitalization rates. The diagnosis and procedure codes utilized to build the cohort and execute the analyses are presented in Supplement 1, available online.

### Statistical Analysis

Relative risks (RRs) and 95% confidence intervals (CIs) were calculated to determine the risk that patients with BPD who underwent facial plastic surgery had specific demographic, surgical, and clinical characteristics and outcomes compared to patients without BPD. A *t* test was performed to determine whether there was a difference in mean age between groups. All statistical analyses were conducted using the TriNetX platform, which is based on Java, R, and Python.

## Results

### Demographics

From 2012 to 2023, a total of 60,762 patients underwent facial plastic surgery, including 309 patients with a diagnosis of BPD (0.51%) and 60,453 controls (Table 1). The mean age of patients with BPD was 45.0 ± 17.1 years, with 77.0% female and 22.0% male. In comparison, controls had a mean age of 54.7 ± 19.8 years, with 63.4% female and 34.5% male. Regarding race, <10 patients in the cohort with BPD identified as American Indian or Alaska Native, Asian, black, or Native Hawaiian or other Pacific Islander, 4.2% and 5.8% identified as other or had an unknown race, respectively, and 84.1% identified as white. Among controls, 0.4% identified as American Indian or Alaska Native, 3.4% as Asian, 3.5% as black, 0.2% as Native Hawaiian or other Pacific Islander, 4.4% and 8.7% as other or unknown, respectively, and 79.4% as white. Regarding ethnicity, 8.4% of patients with BPD identified as Hispanic or Latino, 81.2% as not Hispanic or Latino, and 10.4% had an unknown ethnicity. In the control cohort, 9.4% identified as Hispanic or Latino, 75.0% as not Hispanic or Latino, and ethnicity was unknown for 15.6%. Among patients with BPD, 65.7% (n = 203) had a known diagnosis before the time of surgery, whereas 34.3% (n = 106) were diagnosed postoperatively. Patients with BPD were significantly younger (*P* < .001) and more likely to be female (*P* < .001) compared to those without BPD. They were also significantly more likely to reside in the Midwest (*P* < .001) and to be divorced or never married (both *P* < .001).

**Table 1:** Differences in Demographics Between Patients With Borderline Personality Disorder and Controls Who Underwent Facial Plastic Surgery (n = 60,762)^a^

	Borderline personality disorder (n = 309)	Controls (n = 60,453)	RR (95% CI)	*P*
Age, y	45.0 ± 17.1	54.7 ± 19.8	‐	<.001
Sex				
Female	238 (77.0%)	38,303 (63.4%)	1.22 (1.14‐1.29)	<.001
Male	68 (22.0%)	20,867 (34.5%)	0.64 (0.52‐0.79)	<.001
Unknown	<10 (<3.2%)	1,283 (2.1%)	‐	‐
Race				
American Indian or Alaska Native	<10 (<3.2%)	242 (0.4%)	‐	‐
Asian	<10 (<3.2%)	2039 (3.4%)	‐	‐
Black or African American	<10 (<3.2%)	2104 (3.5%)	‐	‐
Native Hawaiian or other Pacific Islander	0 (0%)	96 (0.2%)	‐	‐
Other	13 (4.2%)	2675 (4.4%)	‐	‐
Unknown race	18 (5.8%)	5233 (8.7%)	‐	‐
White	260 (84.1%)	48,064 (79.4%)	‐	‐
Ethnicity				
Hispanic or Latino	26 (8.4%)	5695 (9.4%)	‐	‐
Not Hispanic or Latino	251 (81.2%)	45,361 (75.0%)	‐	‐
Unknown ethnicity	32 (10.4%)	9397 (15.6%)	‐	‐
Region				
Midwest	79 (25.6%)	11,255 (18.6%)	1.37 (1.13‐1.66)	.001
Northeast	85 (27.5%)	17,626 (29.2%)	0.94 (0.79‐1.13)	.53
Other/unknown	<10 (<3.2%)	989 (1.6%)	‐	‐
South	86 (27.8%)	19,382 (32.1%)	0.87 (0.73‐1.04)	.12
West	58 (18.8%)	11,201 (18.5%)	1.01 (0.80‐1.28)	.91
Marital status				
Divorced	27 (8.7%)	2274 (3.8%)	2.32 (1.62‐3.34)	<.001
Married	33 (10.7%)	12,444 (20.6%)	0.52 (0.38‐0.72)	<.001
Never married	55 (17.8%)	6877 (11.3%)	1.56 (1.23‐1.99)	<.001
Unknown	189 (61.2%)	36,206 (59.9%)	1.02 (0.93‐1.12)	.64
Widowed	<10 (<3.2%)	2652 (4.4%)	‐	‐

Abbreviations: CI, confidence interval; RR, relative risk.

^a^ For demographic outcomes with 1 to 10 people, TriNetX rounds to 10.

### Surgical Characteristics

In terms of surgical intervention, patients with BPD were significantly more likely to undergo rhinoplasty compared to patients who did not have BPD (*P* < .001) (Table 2). These patients were also less likely to undergo blepharoplasty (*P* < .001) or facelift (*P* = .01). There were no significant differences in the rates of browlift (*P* = .59) or lipectomy (*P* = .54) between groups.

**Table 2:** Differences in Surgical and Clinical Characteristics Between Patients With Borderline Personality Disorder and Controls Who Underwent Facial Plastic Surgery (n = 60,762)

	Borderline personality disorder (n = 309)	Controls (n = 60,453)	RR (95% CI)	*P*
Surgery				
Blepharoplasty	96 (31.1%)	28,873 (47.8%)	0.65 (0.55‐0.77)	<.001
Browlift	52 (16.8%)	9507 (15.7%)	1.07 (0.83‐1.37)	.59
Facelift	13 (4.2%)	5086 (8.4%)	0.50 (0.29‐0.85)	.01
Lipectomy	9 (2.9%)	1440 (2.4%)	1.22 (0.64‐2.33)	.54
Rhinoplasty	196 (63.4%)	24,933 (41.2%)	1.54 (1.41‐1.67)	<.001
Medical comorbidities				
Cerebrovascular diseases	81 (26.2%)	10,196 (16.9%)	1.55 (1.29‐1.88)	<.001
Chronic kidney diseases	43 (13.9%)	7173 (11.9%)	1.17 (0.89‐1.55)	.26
Chronic lower respiratory diseases	177 (57.3%)	19,009 (31.4%)	1.82 (1.65‐2.01)	<.001
Hypertensive diseases	161 (52.1%)	30,393 (50.3%)	1.04 (0.93‐1.15)	.51
Ischemic heart diseases	92 (29.8%)	13,008 (21.5%)	1.39 (1.17‐1.64)	<.001
Liver disease	98 (31.7%)	9514 (15.7%)	2.02 (1.71‐2.38)	<.001
Type 2 diabetes mellitus	101 (32.7%)	13,951 (23.1%)	1.61 (1.27‐2.05)	<.001
Psychiatric comorbidities				
Anxiety	286 (92.6%)	21,659 (35.8%)	2.58 (2.50‐2.67)	<.001
Bipolar disorder	142 (46.0%)	1735 (2.9%)	16.01 (14.07‐18.23)	<.001
Delusional disorder	42 (13.6%)	618 (1.0%)	14.32 (10.44‐19.65)	<.001
Depression	277 (89.6%)	19,422 (32.1%)	18.04 (12.52‐26.01)	<.001
Eating disorder	59 (19.1%)	988 (1.6%)	11.68 (9.21‐14.82)	<.001
OCD	52 (16.8%)	778 (1.3%)	13.08 (10.11‐16.92)	<.001
PTSD	142 (46.0%)	2147 (3.6%)	12.94 (11.39‐14.70)	<.001
Self‐harm	70 (22.7%)	732 (1.2%)	18.71 (15.04‐23.27)	<.001
Substance use disorders				
Abuse of other psychoactive substances	102 (33.0%)	3058 (5.1%)	8.98 (7.11‐11.35)	<.001
Alcohol use disorders	102 (33.0%)	5643 (9.3%)	3.54 (3.01‐4.15)	<.001
Cannabis disorders	77 (24.9%)	1480 (2.4%)	10.18 (8.33‐12.43)	<.001
Cocaine abuse	37 (12.0%)	541 (0.9%)	13.38 (9.78‐18.31)	<.001
Inhalant abuse	31 (10.0%)	492 (0.8%)	12.33 (8.73‐17.41)	<.001
Nicotine dependence	156 (50.5%)	10,326 (17.1%)	2.96 (2.64‐3.31)	<.001
Opioid disorders	59 (19.1%)	1483 (2.5%)	7.78 (6.15‐9.84)	<.001

Abbreviations: CI, confidence interval; OCD, obsessive‐compulsive disorder; PTSD, posttraumatic stress disorder; RR, relative risk.

### Clinical Characteristics

Clinically, patients with BPD had significantly higher rates of cerebrovascular diseases, chronic lower respiratory diseases, ischemic heart diseases, liver disease, and type 2 diabetes (all *P* < .001) (Table 2). They also had significantly higher rates of comorbid depression, anxiety, bipolar disorder, posttraumatic stress disorder (PTSD), obsessive‐compulsive disorder, delusional disorders, eating disorders, and a history of self‐harm (all *P* < .001). Furthermore, these patients were also more likely to have a documented substance use disorder, including nicotine dependence, alcohol use disorders, cannabis disorder, opioid disorders, cocaine abuse, inhalant abuse, and abuse of other psychoactive substances (all *P* < .001).

### Postoperative Care and Complications

During the baseline period (9–12 months before surgery), patients with BPD had significantly higher rates of emergency department visits and inpatient admissions compared to controls (both *P* < .001) (Table 3). These elevated rates persisted in the 3 months following surgery, with BPD patients again demonstrating significantly higher emergency department visits and inpatient admissions than controls (both *P* < .001). However, when comparing postoperative health care utilization to baseline within the BPD group, emergency department visit rates remained unchanged (RR 1.00, 95% CI 0.70‐1.44, *P* = 1.0), and inpatient admissions were not significantly different from baseline (RR 1.19, 95% CI 0.76‐1.87, *P* = .44).

**Table 3:** Differences in Baseline Hospitalization Rates, Rehospitalization, General Postoperative Complications, and Revision Surgery Between Patients With Borderline Personality Disorder and Controls Who Underwent Facial Plastic Surgery (n = 60,762)

	Borderline personality disorder (n = 309)	Controls (n = 60,453)	RR (95% CI)	*P*
Baseline hospitalization rate 1 y to 9 mo before surgery				
Emergency department	49 (15.9%)	2341 (3.9%)	4.10 (3.16‐5.31)	<.001
Inpatient admission	31 (10.0%)	1904 (3.1%)	3.19 (2.27‐4.46)	<.001
Rehospitalization 0 to 3 mo				
Emergency department	49 (15.9%)	2907 (4.8%)	3.30 (2.55‐4.27)	<.001
Inpatient admission	37 (12.0%)	3999 (6.6%)	1.81 (1.34‐2.45)	<.001
General postoperative complications 0 to 3 mo				
Bleeding/hematoma	1 (0.3%)	232 (0.4%)	0.84 (0.12‐5.99)	.86
Surgical site infection	0 (0%)	133 (0.2%)	0.73 (0.05‐11.71)	.82
Wound dehiscence	4 (1.3%)	422 (0.7%)	1.85 (0.70‐4.93)	.22
Revision surgery 0 to 1 y				
Blepharoplasty	2/96 (2.1%)	1048/28,873 (3.6%)	0.57 (0.15‐2.26)	.43
Browlift	2/52 (4.8%)	339/9507 (3.5%)	1.04 (0.27‐4.06)	.96
Facelift	0/13 (0%)	193/5086 (3.7%)	0.94 (0.06‐14.33)	.96
Lipectomy	0/9 (0%)	58/1440 (4.0%)	1.23 (0.08‐18.57)	.88
Rhinoplasty	15/196 (7.7%)	1312/24,933 (5.3%)	1.47 (0.90‐2.40)	.13

Abbreviation: RR, relative risk.

The rates of general postoperative complications, such as bleeding/hematoma, surgical site infections, and wound dehiscence, were minimal and did not differ significantly between groups (all *P* > .05). Although patients with BPD who underwent rhinoplasty had a higher rate of revision surgery compared to controls, this difference was not statistically significant (*P* = .13). Revision rates for other procedures, including blepharoplasty, brow lift, facelift, and lipectomy, showed no significant differences between groups (all *P* > .05).

## Discussion

The literature consistently documents a higher prevalence of psychiatric comorbidities, including BPD,^11^ among individuals seeking facial plastic surgery.^19^, ^20^, ^21^ Previous research across various surgical fields has shown that patients with psychiatric conditions are at an elevated risk for postoperative complications.^22^, ^23^, ^24^ In facial plastic surgery, a study by Napoleon reported that patients with BPD had markedly lower postoperative satisfaction scores (1.5/10) compared to those with other personality disorders (5/10) and those without any personality disorders (7.4/10).^10^ Our study extends upon prior research by providing a comprehensive, large‐scale analysis of the demographics, surgical preferences, clinical characteristics, and outcomes for patients with BPD undergoing facial plastic surgery. Overall surgical complication and early revision rates were low. Patients with BPD were more than three times as likely to visit the emergency department and nearly twice as likely to require hospital admission postoperatively; however, these rates were comparable to their preoperative baseline.

The prevalence of documented BPD in this cohort who underwent facial plastic surgery was relatively low, with only 0.51% having a diagnosis. However, it is likely that the true prevalence of BPD is higher, as the condition is underdiagnosed.^25^ Moreover, these data reflect only those who proceeded with surgery, and the number of patients with BPD who present to the clinic seeking surgical consultation is likely much greater. This limits the applicability of our findings to surgical candidacy decisions. It has been estimated that the rate of BPD could be as high as 9% in this population.^10^ Additionally, more than a third of patients in our cohort were not diagnosed with BPD until after surgery, highlighting the importance of screening for BPD and other psychiatric conditions. Early identification can facilitate appropriate psychiatric evaluation and treatment, ensuring comprehensive care.

We found that these patients were, on average, 10 years younger, more frequently female, and more likely to be divorced compared to the general population. BPD symptoms are often identified before adulthood.^26^ This may have contributed to the younger demographic among the BPD cohort compared to controls undergoing surgery and does not necessarily indicate that BPD directly leads individuals to pursue surgical interventions. Although facial plastic surgery is more common among females,^27^ the proportion of female patients with BPD undergoing these procedures was disproportionately high, consistent with earlier findings that BPD is more commonly diagnosed in women.^28^, ^29^ However, recent research suggests that this gender disparity may be influenced by diagnostic biases rather than reflecting actual differences in prevalence.^30^, ^31^ Therefore, facial plastic surgeons should not overlook the potential for a BPD diagnosis in male patients. BPD symptoms vary by gender: females often show emotional instability, interpersonal issues, and comorbid mental health conditions such as mood disorders, PTSD, and eating disorders. Conversely, males are more prone to explosive temperaments, aggression, and substance abuse.^32^ Other indicators that a patient may have BPD include a history of adverse childhood events, previous esthetic procedures with different surgeons, splitting perspectives of clinic staff or past surgeons, and changing bodily concerns over time.^11^ Up to 90% of patients with BPD report a history of traumatic childhood experiences.^33^

Patients with BPD were more than 50% more likely to undergo rhinoplasty and less likely to select blepharoplasty or facelift. Rhinoplasty, being one of the most psychologically impactful surgeries, may attract patients with BPD due to their heightened concerns about self‐image. This preference aligns with findings of another study on patients with BDD, who also favored rhinoplasty.^34^ Such trends reflect the deep‐seated issues with self‐identity and body image prevalent in both BPD and BDD. It is likely that many patients with BPD in our study had comorbid BDD; however, this was unable to be reliably measured given that the ICD‐10 code for BDD (F45.22) was only recently introduced in October 2023. Studies have estimated the prevalence of BDD in the facial plastic surgery setting to be more than 13%.^35^ As more patients are ascribed the BDD ICD‐10 code over time, a similar study could be conducted to further characterize that condition.

There was a high prevalence of medical and psychiatric comorbidities, as well as substance abuse among patients with BPD undergoing facial plastic surgery. Psychiatric conditions and substance use are common in BPD,^36^, ^37^ and likely contributed to the increased rates of emergency department visits and inpatient admissions observed. These elevated rates were present not only during the postoperative recovery period but also during the preoperative baseline. These findings suggest that although BPD is associated with elevated health care utilization, surgery did not appear to amplify this difference beyond the already high baseline. Their elevated health care use may reflect a broader pattern of chronic or baseline health system interaction rather than a disproportionate response to surgery itself. Another possible hypothesis for this is that patients with BPD are already engaged in frequent health care contact, so new surgical issues may be managed within existing patterns of care. Regardless of whether surgery influences postoperative health care utilization for this patient population, there is a need for thorough preoperative assessments and a multidisciplinary approach to address both the physical and psychological health of this population.

Overall, patients with BPD may have difficulty achieving satisfaction with their surgical results due to the inherent characteristics of the condition, such as pervasive instability in mood and self‐image. The impulsivity associated with BPD often leads to rushed decisions about undergoing surgery without thoroughly considering the long‐term consequences, increasing the likelihood of disappointment with the results. True subjective outcomes, such as patient satisfaction, could not be captured in this large database study, which remains a valuable area for future research. However, one notable finding in our analysis of revision surgery was that patients with BPD who underwent rhinoplasty were 1.5 times as likely to undergo revision surgery in the first year, though this did not achieve statistical significance. Our analysis was limited to revision surgeries occurring within 1 year of the index procedure due to sample size constraints; as many revisions take place beyond this period, longer‐term outcomes may not have been fully captured in this cohort. Additionally, if a patient sought further care by a different surgeon at an outside HCO, they would not have been counted as undergoing revision surgery. Therefore, although early reoperation rates offer some insight, they may not fully reflect the broader picture of dissatisfaction in patients with BPD.

The specialty of facial plastic surgery should consider these findings to enhance preoperative screening and patient counseling when determining surgical candidacy to achieve optimal outcomes for both patients and surgeons. Future studies could focus on developing standardized protocols for managing patients with BPD in facial plastic surgery, evaluating the impact of multidisciplinary care teams, and investigating long‐term surgical outcomes and satisfaction in this unique patient population.

This study represents the largest analysis to date of patients with BPD undergoing facial plastic surgery, but it has several limitations. First, the analysis relies on the accuracy of diagnosis and procedure codes documented in medical records, so there is potential for underdiagnosis or misclassification. The database nature of the study limits the level of detail available for individual patients, requiring all BPD diagnoses to be grouped together, despite the condition varying in severity and duration (mild/severe, acute/chronic).^38^ Additionally, we were unable to determine which patients were receiving professional psychiatric treatment. It was also not possible to determine the reason behind each emergency department visit. Another limitation is that TriNetX mainly gathers data from large HCOs, excluding smaller, private, or cosmetic‐only practices from the analysis and thus limiting generalizability. There were also statistically significant differences in demographics and comorbidities between groups which may have impacted outcome data including procedure type and health care utilization; however, we were unable to perform propensity score matching as the BPD patients meeting matching criteria was too small to yield statistically meaningful results. Finally, it was not possible to differentiate between cosmetic and functional procedures using the database, so some noncosmetic cases were likely included. Although this study offers valuable insights into the prevalence, demographics, clinical characteristics, and outcomes of patients with BPD undergoing facial plastic surgery, caution must be taken in interpreting these findings. Ultimately, surgical decisions should be made on an individualized basis, taking into account the full clinical context and unique needs of each patient.

## Conclusions

Patients with BPD seeking facial plastic surgery exhibit distinct demographic profiles, specific surgical preferences, and higher rates of psychiatric comorbidities and substance use disorders compared to the general population undergoing facial plastic surgery.

## Author Contributions

**F. Jeffrey Lorenz**, concept design, data collection, reviewing data analysis, writing manuscript; **Cheng Ma**, concept design, reviewing data analyses, critical editing of manuscript and final approval; **Alyssa K. Givens**, concept design, reviewing data analyses, critical editing of manuscript and final approval; **Scott G. Walen**, concept design, reviewing data analyses, critical editing of manuscript and final approval.

## Disclosures

### Competing interests

The authors declare that there is no conflict of interest.

### Funding source

The project was supported by the National Center for Advancing Translational Sciences, National Institutes of Health (NIH), through Grant UL1 TR002014.

## Supporting information


**Supporting Information**.
