# Supplementary material for: Borderline Personality Disorder Diagnoses in Facial Plastic Surgery: A Large Database Analysis
Source: OTO Open. 2025 May 29;9(2):e70135. doi: 10.1002/oto2.70135 (PMC12121448; doi:10.1002/oto2.70135)
Supplement: Supplementary file 1 — Supporting Information. [file OTO2-9-e70135-s001.docx]

**Supplement 1:** Diagnosis (ICD-10) and Procedure (CPT) Codes

The diagnosis and procedure codes utilized to execute the database query are listed.

Surgeries

Rhinoplasty: 1005721 (includes 30400, 30410, 30420), 1005725 (includes 30430, 30435, 30450), 30465

Rhytidectomy: 1003504 (includes 15824, 15825, 15826, 15828, 15829)

Brow lift: 67900

Blepharoplasty: 1014047 (includes 15820, 15821), 1014048 (includes 15822, 15823)

Lipectomy of head or neck: 15838, 15876

Medical Comorbidities

Cerebrovascular diseases: I60-I69

Chronic kidney disease: N18

Chronic lower respiratory diseases: J40-J4A

Hypertensive diseases: I10-I1A

Ischemic heart diseases: I20-I25

Liver disease: K70-K77

Type 2 diabetes mellitus: E11

Psychiatric and Substance Use Disorders

Borderline personality disorder: F60.3

Depression: F32

Anxiety disorder: F41

Bipolar disorder: F31

Posttraumatic stress disorder: F43.1

Obsessive-compulsive disorder: F42

Delusional disorder F22

Eating disorder: F50

History of self-harm: Z91.5

Nicotine dependence: F17

Alcohol use disorders: F10

Cannabis disorders: F12

Opioid disorders F11

Cocaine abuse: F14

Inhalant abuse: F18

Abuse of other psychoactive substances: F19

Complications

Postprocedural bleeding/hematoma: L76.2, L76.3, L76.8

Wound dehiscence: T81.3

Surgical site infection: T81.41, T81.42, T81.49
